# Comment on Lahoud et al. Burden and Mortality Outcomes of *Clostridioides difficile* Infection Among Patients with Chronic Obstructive Pulmonary Disease: Findings from a Nationwide Database. *J. Clin. Med.* 2026, *15*, 4110

**DOI:** 10.3390/jcm15176802

**Published:** 2026-09-02

**Authors:** Brett D. Cohen

**Affiliations:** Department of Internal Medicine, St. Luke’s University Health Network, Bethlehem, PA 18015, USA; brett.cohen@gmail.com

We read with interest the study by Lahoud et al. [1], which used the National Inpatient Sample to compare in-hospital outcomes of CDI in patients with and without COPD. We wish to raise a methodological issue that affects the study’s central, population-level claims.

The Methods section contains a direct internal contradiction regarding the survey design. Section 2.3 states that “discharge weights were not used in this study,” whereas Section 2.4 reports that the analysis “utilized the complex sampling capabilities of” SPSS and applied the “second-order Rao-Scott Chi-Square Test.” These statements cannot both be correct: the Rao–Scott correction and complex-samples estimation are defined by the survey design (discharge weight, stratum, and cluster) and cannot be implemented if discharge weights were not applied.

The distinction is not academic. The NIS is an approximately 20% sample of US discharges, and nationally representative estimates are obtained only by applying the discharge weight (DISCWT) within the survey design (strata NIS_STRATUM, clusters HOSP_NIS) [2]. If, as Section 2.3 states, weights were not used, then the figure of 290,172 patients is an unweighted sample count, not the “nationally representative” total the paper describes, and the statements about national burden are not supported. The magnitude of this figure supports that reading: had the discharge weight (approximately five) been applied, the national estimate would be several-fold larger, so a study population reported at sample scale indicates that weights were not in fact applied. If instead a complex-samples analysis was performed (Section 2.4), then the design is described inconsistently and cannot be reproduced. Under either interpretation, the standard errors are mis-specified: ignoring weights, strata, and clustering biases variance estimates, and therefore the confidence intervals and *p*-values on which the reported associations (for example, in-hospital mortality odds ratio 1.346) depend.

The within-cohort comparison of COPD versus no COPD may be less sensitive to weighting than the absolute burden figures; nonetheless, valid inference and any nationally scaled statement require the full survey specification. We encourage the authors to clarify which procedure was actually performed and, if necessary, to re-estimate the models using the HCUP-recommended specification, svyset HOSP_NIS [pweight = DISCWT], strata (NIS_STRATUM), so that the burden and precision claims can be interpreted as intended.

This issue does not diminish the clinical relevance of CDI in COPD, but it determines whether the study’s national and inferential claims can stand as written.

## Data Availability

No new data were created or analyzed in this study. Data sharing is not applicable to this article.
